# Although it's painful: The importance of stringent antibody validation

**DOI:** 10.1371/journal.ppat.1006701

**Published:** 2018-01-04

**Authors:** Victoria A. Meliopoulos, Stacey Schultz-Cherry

**Affiliations:** Department of Infectious Diseases, St. Jude Children’s Research Hospital, Memphis, Tennessee, United States of America; University of Pittsburgh, UNITED STATES

The process of making antibodies is costly and time-consuming. Commercial antibodies offer a convenient solution. However, recent concerns have resulted in a National Institutes of Health (NIH) mandate to vigorously test the specificity of antibodies used in publications (http://grants.nih.gov/reproducibility). Currently, there is no standard for validation and reference data that must be provided in publications [1, 2], and crucial specificity data are often unavailable. Multiple studies have focused on issues of antibody specificity towards proteins such as G-protein-coupled receptors, kinase receptors, and integrins [3–5]. To determine whether an antibody is suitable, the following three issues must be considered: ability to detect the target (specificity), detection of the target above background (sensitivity), and generation of consistent results (reproducibility). Sensitivity is especially problematic with low-abundance proteins, for which the antibody in question can only detect high levels of target.

Problems with reproducibility often arise due to lot-to-lot variability and affect both polyclonal (pAbs) and monoclonal antibodies (mAbs). pAbs are a heterogeneous mixture of antibodies that recognize multiple epitopes of the same target protein but can also include nonspecific antibodies. Each lot, even when prepared from the same donor animal, contains diverse antibody clones and concentrations [6]. However, it is possible to reduce nonspecific binding of a pAb via immunoaffinity enrichment [7]. mAbs, although generally more consistent, are not exempt from variation. Hybridomas maintained in ascites can be contaminated with endogenous immunoglobulins and other proteins, especially if the mAb is not purified [8]. A hybridoma might also lose its antibody gene through continued passaging [7]. Additionally, epitopes that mAbs target are generally short sequences of amino acids that might exist on other proteins [8]. In one report, an mAb targeting the Met tyrosine kinase receptor—a marker of breast cancer diagnosis—revealed the target protein in the nucleus, while another lot showed membrane and cytoplasmic staining [5].

Our laboratory recently published a study focused on β6 integrin (β6), a small heterodimeric molecule involved in cell signaling [9]. The specificity of antibodies to receptors and proteins implicated in cell signaling is not well defined in the literature [6]. It is especially difficult to generate antibodies against specific integrins due to their similar structures [9], and many specification sheets report a small degree (10%–20%) of cross-reactivity with other integrins and proteins [3]. In our work, we purchased several commercial antibodies from different companies to evaluate specificity.

**Fig 1:**
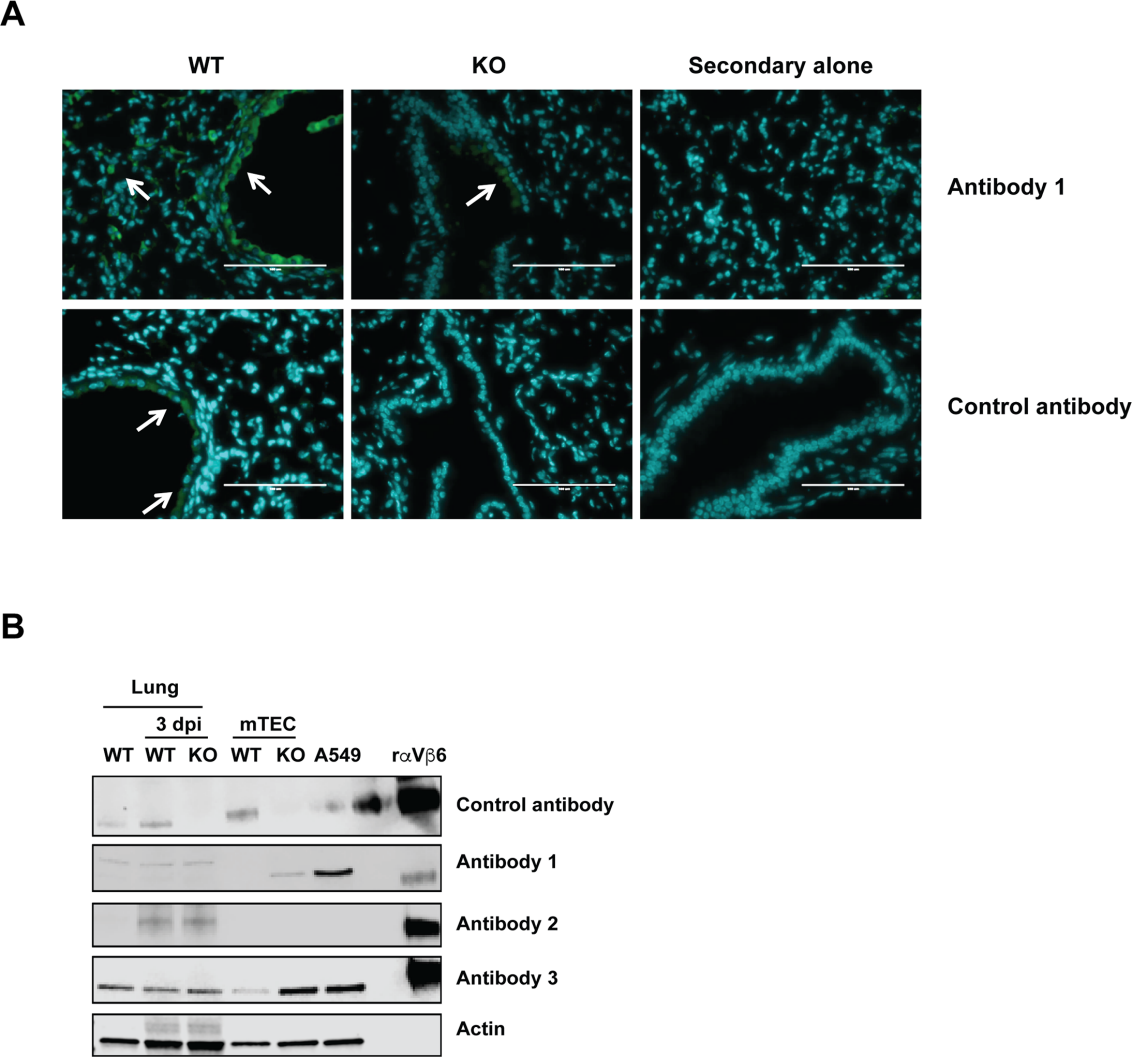
Specificity of β6 antibodies in immunofluorescent microscopy and western blot analysis. (A) Mouse lung sections collected at 3 dpi were stained for β6. Arrows indicate areas of β6 expression. Green = β6; blue = nuclei. Bar is 100 μm. (B) Lysates prepared from lung tissue or tracheal epithelial cells were immunoblotted to detect β6 by using a panel of antibodies. β6, β6 integrin; dpi, days post infection; KO, knockout; mTEC, murine tracheal epithelial cell; WT, wild type.

We used antibody 1 for immunofluorescence (IFA) staining of mouse pulmonary tissues to detect β6. Initially, we detected a strong signal by IFA. However, we also detected a weaker yet concerning signal in confirmed β6 knockout (KO) mice (Fig 1A). Slides stained with only secondary antibody were negative, suggesting that the signal was not due to nonspecific binding of the secondary antibody. Fortunately, we received a highly specific antibody from a collaborator (control antibody) and stained tissues successfully without detecting signal in sections from KO mice.

Western blots presented a similar problem. Lysates from whole lung were collected at 3 days post infection (dpi), a time when β6 is expressed in response to damage but before the infection has resulted in sloughing of β6^+^ epithelial cells [10]. Lysates of infected murine tracheal epithelial cells (mTECs), A549 cells (human lung adenocarcinoma cells, which highly express β6 and are often used as a positive control for antibodies that cross-react with human β6 [11]), as well as recombinant αVβ6 integrin as a positive control, were separated under reducing and nonreducing conditions with a panel of commercially available anti-β6 antibodies (Fig 1B). The control antibody detected more β6 at 3 dpi in infected wild-type (WT) lung compared with uninfected WT lung and detected β6 in WT mTECs but not in KO mTECs or KO lung. A faint band was also detectable in A549 lysate. However, antibody 1 detected bands in all 3 lung samples and, oddly, in KO but not WT mTECs. Surprisingly, antibody 2, which detects human β6, could detect bands in WT and KO lung homogenate but not in mTECs or A549. Antibody 3 detected strong signal in all samples tested. All antibodies easily detected recombinant αVβ6.

We chose antibody 3 to determine what was being detected in the KO samples. We performed sodium dodecyl sulfate polyacrylamide gel electrophoresis (SDS-PAGE) to separate lysates from KO lung and KO mTECs and excised the protein band corresponding with the band detected by antibody 3 to identify the proteins by mass spectrometry. Nine proteins were present in both samples at a threshold of 10 spectral counts or greater (Table 1). Although these data do not confirm that antibody 3 is binding to these proteins, their presence suggests they are likely candidates. However, it is also possible that the proteins are simply of a similar molecular weight.

**Table 1:** Proteins present in area recognized by antibody 3.

Gene	Protein	Mass (kDa)
**ACTN4**	Alpha-actinin-4	105
**EEF2**	Elongation factor 2	95
**GSN**	Gelsolin	86
**HSP1AB**	Heat shock 70 kDA protein 1B	70
**HSP90AA1**	Heat shock protein HSP 90-alpha	85
**HSP90AB1**	Heat shock protein HSP 90-beta	83
**MSN**	Moesin	68
**PLS3**	Plastin-3	71
**PKM**	Pyruvate kinase PKM	58 (dimerized)

Our experiences highlight the importance of validating reagents even if obtained from commercial sources. Not only could nonspecific antibodies compromise research, there could be serious consequences leading to misdiagnoses or to incorrect or delayed treatments when they are used for clinical screenings. Consider that β6 itself is an important biomarker in the clinical setting, often confirming disease. The presence of β6 in tissue sections is used as an indicator for many types of cancer, including cholangiocarcinoma [12], malignant epithelial ovarian cancer [13], breast cancer [5, 14], and pancreatic cancer [15]; it is also a determinant of the metastatic potential of thyroid cancer [16]. β6 is also used as a diagnostic agent for foot and mouth disease virus due to its ability to bind and identify all representative serotypes of the virus in diagnostic ELISAs [17]. Nonspecificity could have a significant impact on the conclusions drawn from clinical trials and lead to incorrect diagnosis [14, 15, 18–20]. These problems are highlighted in several publications [21–24].

The solution is stringent validation. Several groups have published their own laboratory’s antibody validation workflow process (summarized in [6]). Most agree that the first step is a western blot using multiple cell lines or tissues known to express the protein of interest [6, 25]. As we and others have discovered, it is advantageous to use a KO animal or cell line to demonstrate specificity [25]. We would not have known there was a problem with antibody 1 if we had not used KO mice as a control. However, because not all proteins have KO models, it is beneficial to test the antibody in cells in which the target protein has been silenced by RNA interference, for example [25]. Cells transfected to overexpress the target or multiple antibodies binding to different epitopes of the target protein are useful positive controls [25]. A specific antibody should also show a titration effect when the samples or the antibody itself is diluted. Ask the following questions: is the antibody only detecting protein in cell lines expected to express that protein? Are staining patterns similar when different antibodies to the same target are used? Is protein abundance between those antibodies congruent?

As an additional resource, www.antibodypedia.com provides a repository of validation data that is searchable by target. Similar databases are available (summarized in [7]), but the generation of one standardized repository of information would be useful. Do not rely on the literature. Many journals have strict space limitations, and the methods section is often truncated, omitting validation controls. Journals have inconsistent requirements for information supplied, such as clone number, lot number, or dilution used. For example, knowing the exact lot used in the publication could help with reproducibility issues in other labs. Western blots are commonly cropped to show only the band of interest, omitting any nonspecific bands; however, seeing the full data can help researchers troubleshoot antibody-based assays and avoid wasting research funding on repeating unreported data [7]. In fact, many journals are pushing towards standard requirements to report details of antibodies used [1]. Because new research depends upon previously published data, the onus is on every researcher to properly validate tools.
